# Clustering in surgical trials – database of intra-cluster correlations

**DOI:** 10.1186/1745-6215-12-S1-A24

**Published:** 2011-12-13

**Authors:** Jonathan A Cook, Thomas Bruckner, Graeme S MacLennan, Christoph M Seiler

**Affiliations:** 1 Health Services Research Unit, University of Aberdeen, Aberdeen, AB25 2ZD, UK; 2 Institute of Medical Biometry and Informatics, University of Heidelberg, 69120, Heidelberg, Germany; 3 Department of General, Visceral and Trauma Surgery, University of Heidelberg, Heidelberg, 69120, Germany

## Background

Randomised trials evaluating surgical interventions are often design and analysed as if the outcome of individual patients is independent of the surgeon providing the intervention. There is reason to expect outcomes for patients treated by the same surgeon to be more similar than those under the care of another surgeon due to previous experience, individual practice, training, and infrastructure. Such a phenomenon is referred to as the clustering effect and potentially impacts on the required sample size. This depends upon the design and analysis adopted. However, trialists have little data upon which to assess the impact and base trial design. The aim of this study was to quantify the clustering effect by producing a database of surgical trial ICCs.

## Methods

Intra-cluster correlation coefficients (ICCs) were calculated for outcomes from a set of 10 multicentre surgical trials for a range of outcomes and different time points for clustering at both the centre and surgeon level.

## Results

ICCs were calculated for 198 outcomes across the 10 trials at both centre and surgeon cluster levels. The number of cases varied from 138 to 1370 across the trials. The median (range) average cluster size was 32 (9 to 51) and 6 (3 to 30) for centre and surgeon levels respectively. ICC estimates varied substantially between outcomes though uncertainty around individual ICC estimates was substantial. Full details are available online [http://www.abdn.ac.uk/hsru/research/research-tools/study-design].

## Conclusions

Our data for multicentre trials of surgical interventions suggests clustering of outcome is more of an issue than has been previously acknowledged. This database provides trialists with valuable information to aid the design of surgical trials. We anticipate that over time the addition of ICCs from further surgical trial datasets will enhance the usefulness of the database.

